# Dialysis Procedures Alter Metabolic Conditions

**DOI:** 10.3390/nu9060548

**Published:** 2017-05-27

**Authors:** Bernd Stegmayr

**Affiliations:** Department of Public Health and Clinical Medicine, Umea University, 90187 Umea, Sweden; bernd.stegmayr@medicin.umu.se

**Keywords:** malnutrition, lipids, lipase, heparin, dialysis, hemodialysis, peritoneal dialysis, glycation end products, glucose

## Abstract

A progressive chronic kidney disease results in retention of various substances that more or less contribute to dysfunction of various metabolic systems. The accumulated substances are denominated uremic toxins. Although many toxins remain undetected, numerous newly defined toxins participate in the disturbance of food breakdown. In addition, toxic effects may downregulate other pathways, resulting in a reduced ability of free fatty acid breakdown by lipoprotein lipase (LPL) and hepatic lipase (HL). Dialysis may even worsen metabolic functions. For LPL and HL, the use of heparin and low molecular weight heparin as anticoagulation during hemodialysis (HD) initiate a loss of these enzymes from their binding sites and degradation, causing a temporary dysregulation in triglyceride breakdown. This lack of function will cause retention of the triglyceride containing lipids for at least 8 h. In parallel, the breakdown into free fatty acids is limited, as is the energy supply by them. This is repeated thrice a week for a normal HD patient. In addition, dialysis will cause a loss of amino acids and disturb glucose metabolism depending on the dialysates used. The addition of glucose in the dialysate may support oxidation of carbohydrate and the retention of Amadori products and subsequent tissue alterations. To avoid these effects, it seems necessary to further study the effects of anticoagulation in HD, the extent of use of glucose in the dialysate, and the supplementation of amino acids.

## 1. Chronic Kidney Disease (CKD)

A progressive CKD results in retention of various substances that end up in uremia [1,2,3] and a need of dialysis or transplantation for survival. Substances may, more or less, contribute to dysfunction of various metabolic systems [4]. Accumulated substances are denominated uremic toxins [1]. The number of toxins exceeds 1400, and many of them are still unraveled [5]. 

When the glomerular filtration rate is reduced to less than 15 mL/min, the retention of urea and other toxins may lead to a reduced appetite [6]. In this situation, a protein restriction will to some extent reduce the concentration of metabolites as urea and reduce uremic symptoms [7]. At this stage of chronic kidney disease (CKD), the intestinal flora is altered. Fermentation of protein and amino acids by gut bacteria generates excess amounts of potentially toxic compounds such as ammonia, amines, thiols, phenols, and indoles. In addition, the generation of short-chain fatty acids is reduced. An impaired intestinal barrier function in patients with CKD permits translocation of gut-derived uremic toxins into the systemic circulation. These uremic toxins contribute to the progression of CKD, cardiovascular disease, insulin resistance, and protein-energy wasting [8]. In parallel, the new intestinal flora may result in bowel dysfunction, with altered stools and worsened appetite. 

Progressive weight loss is an important sign of severe kidney dysfunction, and it is an important indicator of dialysis treatment onset if this negative trend of malnutrition is to be broken [6].

## 2. Dialysis

The initiation of adequate doses of dialysis are recommended to limit the side effects of uremic toxins. For hemodialysis (HD), at least three sessions per week and a total time of at least 12 h/w should result in a weekly clearance, eKt/V, of more than 1.2 (for women and patients with high comorbidity 1.4) [9]. 

Dialysis will only partly lower increased levels of various uremic toxins. When performing HD, the lowering of uremic toxins is intermittent for a few hours and thereafter followed by a progressive retention until the next HD [9]. During continuous ambulatory peritoneal dialysis (CAPD), the removal of toxins proceeds through the peritoneal membrane. The efficacy of the peritoneal membrane (clearance) is much lower than a hemodialysis membrane. Thereby, the clearance of urea is approximately 4 mL/min [10,11], whereas during intermittent HD the removal rate is 50 times higher (more than 200 mL/min) [12]. Continuous, everyday treatment, usually for 24 h/day, compensates for limitations in peritoneal transfer. 

Malnutrition, appetite loss, and protein wasting is common despite dialysis treatment and is reported to be present in up to 47% of patients [13,14]. Moreover, besides variables that indicate the presence of inflammation, as increased IL-6 and CRP levels, the dialysis is by itself associated with protein-energy wasting [15]. Protein-energy wasting (PEW) refers to the loss or reduced amounts of body protein mass and fuel reserves [16]. Too extensive fluid intake and thus inter dialytic weight gain (IDWG) [17] and inflammatory processes are variables that interfere with appetite and thus with food intake [6]. In addition, the taste acuity is impaired in uremic and dialysis patients [18]. The appetite and taste acuity is similarly reduced in HD and peritoneal dialysis (PD) patients [15,18]. However, when inflammation is added, it gets worse. Inflammatory episodes are more frequent with HD than they are with PD [15].

## 3. Triglycerides and Hemodialysis

When malnutrition appears, an increase in protein and calories is often recommended to adjust for the lack of energy [6]. Fat contributes to 40% of calories, which derive mainly from triglycerides (TrG) [19]. Triglycerides are transported in blood in the core of lipoproteins (chylomicrons and VLDL). Fatty acids (5% by glycerol) contribute to 95% of that energy content. Lipoprotein lipase (LPL) and hepatic lipase (HL) are key enzymes in the breakdown of triglycerides [20,21,22].

Hyperlipidemia, especially the increase of TrG in all density classes, is present in patients with severe CKD and those on dialysis [6,23,24]. A limitation in lipid breakdown is present due to the reduced amount and partly inhibited function of lipases [20,22,25,26,27]. The lack of functioning lipase will result in an increased concentration of TrG in the blood. The function of lipases is reduced by increased levels of the enzyme inhibitors angiopoietin-like protein 3, 4, and 8 (ANGPTL) [22,28,29,30]. These hepatically derived factors inhibit LPL activity differently. Zhang suggested an ANGPTL3-4-8 model where the ANGPTL8 activates ANGPTL3, in an endocrine manner, to inhibit the activity of LPL activity in the heart and skeletal muscle. On the contrary, ANGPTL4 inhibits LPL activity in white adipose tissue [30]. Fasting upregulates ANGPTL4 (inhibiting LPL in fat tissue) and downregulates ANGPTL8, thereby stimulating LPL in heart and sceletal muscle. While ANGPTL8 is not elevated in HD patients [31], ANGPTL3 and ANGPTL4 are significantly increased [29,32]. Hemodialysis did not eliminate ANGPTL3, while a high flux dialysis filter is necessary to cause a reduction in ANGPTL4 levels [29]. Besides inhibiting factors to LPL, HD patients have only approximately half of the body store of lipases compared with healthy age-matched persons [33]. The body store can be estimated by an intravenous bolus dose of heparin that releases most of the LPL and hepatic lipases from their binding sites [34]. A peak value of the lipase or area under the curve (AUC) can be used for a comparison between groups [33]. The reduction of LPL, upon heparin injection, results in a limitation of breakdown capacity of TrG into FFA and energy. In addition, during HD, in most centers, unfractionated heparin (UFH) and low molecular weight heparin (LMWH) is used in anticoagulation of the extra corporeal circuit to avoid clotting and to maintain an open dialysis circuit [35]. The UFH and LMWH will enter the blood of the patient. Here, UFH and LMWH will immediately induce the HL and LPL to lose their binding from the sites at the endothelia, where they are normally attached, and get distributed throughout the blood [20,34,36]. The release from the binding sites, within minutes, will cause an increase in LPL and HL in the circulation, as well as a subsequent temporary breakdown and reduction of triglycerides [37]. The increased lipase activity due to the enzyme now becomes fully exposed to triglycerides in the circulation. In parallel, the liver will rapidly degrade the released LPL and HL, this degradation is differing between unfractionate heparin and low molecular weight heparins [36,37]. This increased degradation will only slowly be compensated for by new enzyme production. It will take at least 8 h to achieve a recovery to baseline capacity [38,39]. The effect will be that the triglycerides will rise significantly during the end of HD. At that stage TrG will not be degraded into FFA and energy in the extent that the body may find necessary for optimal energy supply, especially if the patient intends to be physically active. A metabolic starvation based on lowered capacity of lipid breakdown will be present. In parallel, enzyme inhibitors angiopoietin-like proteins 3 and 4 are at high levels in the blood [29]. Since HD is usually repeated at least thrice a week for a normal HD patient, an intermittent disturbance of the lipase system will occur. To avoid the disturbances of the lipases (caused by heparin), it seems necessary to clarify other options of anticoagulation such as regional citrate anticoagulation. By using solely citrate (and not UFH), there is no release of lipase from its endothelial bindings sites [29]. If heparin is added, an instant release of the lipases occurs. However, the citrate anticoagulation technique has still not been fully developed for chronic dialysis, using it neither as dialysate [40] nor as regional administration [35]. At present, the regional citrate dialysis requires more surveillance during HD, especially to secure from deviance from normal values of ionized calcium and magnesium [41,42]. 

During CAPD, the decreased levels of lipase will partly impair the ability to degrade lipids. However, these patients do not get UFH or LMWH intravenously thrice a week; thereby, the periods of extensive dysfunction of the lipid breakdown system will not appear. 

To further clarify these changes, it is important to perform long-term controlled studies with or without reduced doses of intravenous UFH or various types of LMWH as anticoagulation to investigate the possibility of preserving the LPL function at its binding sites.

## 4. Protein and Dialysis

The protein breakdown into energy is especially disturbed during inflammation. Thereby, protein-energy wasting (PEW) has numerous causes such as anorexia and decreased food intake caused by uremic toxins, inflammation, and superimposed illnesses. In addition, resistance to anabolic hormones, increased oxidant levels, decreased antioxidants, acidemia, and physical deconditioning are important factors to consider [6]. Further low-flux HD causes a loss of 1–2 g of protein through the dialyzer, whereas, when high flux membranes are used, the loss of 10 g of amino acids in postprandial patients has been reported [43,44,45]. Approximately, 3 g/day of free amino acids are removed during CAPD, and 9 g/day of protein and approximately 5.7 g/day of albumin is lost into the dialysate [46], which can rise up to more than 15 g/day when peritonitis is present [46]. Acidemia can promote protein degradation and aggravate the metabolic disorder [6].

## 5. Carbohydrates and Hemodialysis

Another important energy supply in the dialysis patients is the carbohydrate intake. When the lipase function is impaired, the patients have to rely on glucose metabolism. Therefore, the dietary preference might be of the patient to nourish themselves so as to ingest more carbohydrates. An increased intake of carbohydrates and fat is recommended by dieticians when kidney function is severely impaired (clearance <15 mL/min) and when a protein-restricted diet is prescribed. However, at this stage the body is limited in its carbohydrate metabolism and in addition insulin resistance is usually present [8,47,48,49,50]. 

When performing CAPD, the patients will be loaded with glucose, due to the transfer of glucose from the PD fluid into the vessels of the abdominal cavity. From there, it diffuses into the body. In normoglycemic individuals, approximately 15–25 g of glucose (200–480 kcal of energy) may be removed during HD when glucose free dialysate is used [45,51]. With glucose containing hemodialysate of 200 mg/dL (10 mmol/L), there is, on the contrary, a net absorption of 10–12 g of glucose/HD [6].

During HD, in most European centers, the dialysis fluid will contain glucose at a final concentration of approximately 5 mmol/L. The initial reason for using the glucose within the dialysate is partly to avoid hypoglycemia in patients suffering from diabetes mellitus. However, nowadays, glucose in the dialysate is considered a beneficial supply of energy to the patients.

However, the glucose load from dialysate during HD, especially when using 200 mg/dL [49], will disturb metabolism and contribute to oxidative stress and increased blood glucose and serum insulin [49,52]. The increased levels of oxidative radicals induce oxidation of carbohydrates. The oxidation induces glycation, then the retention of Amadori products, and subsequently tissue damage. In short, reactive derivatives from non-enzymatic glucose–protein condensation reactions, as well as lipids and nucleic acids exposed to reducing sugars, form a heterogeneous group of irreversible adducts called AGEs (advanced glycation end products). The glycation process begins with the conversion of reversible Schiff base adducts to more stable, covalently bound Amadori rearrangement products. Over the course of days to weeks, these Amadori products undergo further rearrangement and condensation reactions to form irreversibly cross-linked macroprotein derivatives known as AGEs [50]. A method of estimating retained glucose degradation products within the body is the skin-autofluorescence device (SAF) [53]. There is a strong correlation between the finding of high values of SAF and the risk for premature death and especially due to cardiovascular causes [54]. The impaired kidney function per se will cause retention of Amadori products and rearrangements of tissue, causing more or less cardiovascular alterations [53]. HD can be performed using a dialysis membranes with either smaller or larger pores. With a dialysate containing glucose (5 mmol/L), and HD with either low flux or high flux (larger pores) dialyzers, there was no change measured by SAF [55,56]. In contrast, a significant reduction of SAF was measured when performing HD without dialysate glucose [57]. Data from high flux HD even indicated the presence of a more extensive backfiltration of glucose through the dialyzer membrane into the blood. This induces subsequent oxidative stress and the presence of higher concentrations of protein-bound glucose degradation products in plasma compared to when HD was performed using low-flux membranes [56]. Therefore, these data question a benefit of glucose containing dialysates to compensate for the dysfunction of lipases for energy supply by TrG breakdown during standard HD. However, glucose containing dialysates may prevent severe hypoglycemia in patients with labile insulin-dependent diabetes mellitus [58]. To investigate if an improved net balance of glycation degradation products can be achieved over the long term, further studies with low glucose or glucose-free dialysate are recommended. 

For CAPD patients, the disadvantage is the extensive glucose load in the dialysis bags, which is continuous throughout the day, unless automated cyclic peritoneal dialysis is used for only part of the day. This may be reflected by the worse skin autofluorescence (SAF) findings in PD versus HD patients, when adjusted for age and dialysis vintage in adults [59], but not different in children [60]. Besides cardiovascular morbidity and increased mortality, high SAF was related to a greater risk for sepsis-related mortality in PD patients [61]. Negative factors for a high SAF were long dialysis vintage and cumulative glucose exposure [59,62]. Patients using icodextrin (Baxter Inc., Deerfield, IL, USA), instead of glucose, as a dialysate had worse SAF than those using glucose only [63]. An indication for the possible reversibility is the finding that patients transplanted had SAF similar to patients with chronic kidney disease stage 3 and less than those on HD and PD [64].

## 6. Consideration of Diet for Dialysis Patients

When confronted with the patient therapeutic strategies to prevent and treat protein-energy wasting in maintenance dialysis, patients are to optimize the dialysis and limit the inflammatory processes. A general investigation should include the evaluation of body size, nutrient intake, and protein-energy wasting with the periodic help of dietary counseling and monitoring of nutrient intake and protein-energy status. The use of inter dialytic weight gain can help to guide patient nutrition and compliance [6,17]. Patients on HD, with heparin as anticoagulation, may benefit from limitation in intake of larger amounts of fat during the HD procedure per se and a few hours thereafter, especially if they suffer from increased blood levels of TrG. Risk of hypoglycemia or presence of severe malnutrition has to be taken into account when removal of glucose in the dialysate is considered. When glucose is used, a concentration of 5 mmol/L seems preferable to 10 mmol/L. Further studies have to clarify whether a lower dialysate glucose concentration or total withdrawal should be compensated for by a protein- and glucose-enriched diet. Patients randomized to receive inter dialytic parenteral nutrition (IDPN), in comparison to those not receiving IDPN, did not show, over two years, improved mortality or hospitalization rates, nor reduced evidence for PEW [65]. Lack of difference may be due to the fact that almost all of the patients received an oral nutritional supplement as well. The use of oral supplement may alone improve whole-body and skeletal muscle protein anabolism in a dose-dependent manner in chronic HD patients [65,66].

The periods between HD seem metabolically optimal for food intake and exercise, since the lipase restores within approximately 4 h after HD. Guidelines recommend intake of approximately 1.2 g/kg/day 50% high biologic value protein and 35 kcal/kg/day (fat 25–35% of total energy intake, <7% saturated fat), unless older than 60 years and/or those with limited exercise or obese [6]. 

Since the HD per se will induce protein catabolism [67], the use of low and high flux dialyzers may further contribute to the loss of amino acids [68,69,70,71,72]. The HD procedure, metabolic disturbance, and reduced extent of exercise can well explain that the working capacity of HD patients is extensively reduced compared to age-matched persons without chronic kidney disease [73]. 

For CAPD patients, there might be a benefit calculating the PD-glucose adsorbed/day by peritoneal transfer through the membrane. This glucose load can help to adjust the content of various nutrients for the daily intake. Adding one amino acid bag instead of glucose reduces the glucose load [6]. This change into an amino acid bag may help to limit protein losses by dialysis [74,75]. 

For patients with diabetes mellitus the risk for hypo- and hyperglycemia needs to be considered [76], and it can well motivate to maintain a glucose containing dialysate in these patients. 

## References

[1] Vanholder R., Argiles A., Baurmeister U., Brunet P., Clark W., Cohen G., De Deyn P.P., Deppisch R., Descamps-Latscha B., Henle T. (2001). Uremic toxicity: Present state of the art. Int. J. Artif. Organs.

[2] Vanholder R., De Smet R., Glorieux G., Argiles A., Baurmeister U., Brunet P., Clark W., Cohen G., De Deyn P.P., Deppisch R. (2003). Review on uremic toxins: Classification, concentration, and interindividual variability. Kidney Int..

[3] Duranton F., Cohen G., De Smet R., Rodriguez M., Jankowski J., Vanholder R., Argiles A., European Uremic Toxin Work Group (2012). Normal and pathologic concentrations of uremic toxins. J. Am. Soc. Nephrol..

[4] Vanholder R., Schepers E., Pletinck A., Nagler E.V., Glorieux G. (2014). The uremic toxicity of indoxyl sulfate and p-cresyl sulfate: A systematic review. J. Am. Soc. Nephrol..

[5] Weissinger E.M., Kaiser T., Meert N., De Smet R., Walden M., Mischak H., Vanholder R.C. (2004). Proteomics: A novel tool to unravel the patho-physiology of uraemia. Nephrol. Dial. Transplant..

[6] Kopple J.D., Shah A., Kalantar-Zadeh K., Lerma E.V., Weir M.R. (2017). Malnutrition and Intradialytic Parenteral Nutrition in End-Stage Kidney Disease Patients. Principles and Practice of Dialysis.

[7] Alvestrand A., Ahlberg M., Furst P., Bergstrom J. (1983). Clinical results of long-term treatment with a low protein diet and a new amino acid preparation in patients with chronic uremia. Clin. Nephrol..

[8] Ramezani A., Massy Z.A., Meijers B., Evenepoel P., Vanholder R., Raj D.S. (2016). Role of the Gut Microbiome in Uremia: A Potential Therapeutic Target. Am. J. Kidney Dis..

[9] Kuhlmann M.K., Kotanko P., Levin N.W., Johnson R.J., Feehally J., Floege J. (2015). Hemodialysis: Outcome and Adequacy. Comprehensive Clinical Nephrology.

[10] Wolfson M., Piraino B., Hamburger R.J., Morton A.R., Icodextrin Study Group (2002). A randomized controlled trial to evaluate the efficacy and safety of icodextrin in peritoneal dialysis. Am. J. Kidney Dis..

[11] Plum J., Gentile S., Verger C., Brunkhorst R., Bahner U., Faller B., Peeters J., Freida P., Struijk D.G., Krediet R.T. (2002). Efficacy and safety of a 7.5% icodextrin peritoneal dialysis solution in patients treated with automated peritoneal dialysis. Am. J. Kidney Dis..

[12] Revaclear Dialyzer Technology. www.baxter.com.

[13] Carrero J.J., Qureshi A.R., Axelsson J., Avesani C.M., Suliman M.E., Kato S., Barany P., Snaedal-Jonsdottir S., Alvestrand A., Heimburger O. (2007). Comparison of nutritional and inflammatory markers in dialysis patients with reduced appetite. Am. J. Clin. Nutr..

[14] Carrero J.J., Nakashima A., Qureshi A.R., Lindholm B., Heimburger O., Barany P., Stenvinkel P. (2011). Protein-energy wasting modifies the association of ghrelin with inflammation, leptin, and mortality in hemodialysis patients. Kidney Int..

[15] Snaedal S., Qureshi A.R., Lund S.H., Germanis G., Hylander B., Heimburger O., Carrero J.J., Stenvinkel P., Barany P. (2016). Dialysis modality and nutritional status are associated with variability of inflammatory markers. Nephrol. Dial. Transplant..

[16] Fouque D., Kalantar-Zadeh K., Kopple J., Cano N., Chauveau P., Cuppari L., Franch H., Guarnieri G., Ikizler T.A., Kaysen G. (2008). A proposed nomenclature and diagnostic criteria for protein-energy wasting in acute and chronic kidney disease. Kidney Int..

[17] Holmberg B., Stegmayr B.G. (2009). Cardiovascular conditions in hemodialysis patients may be worsened by extensive interdialytic weight gain. Hemodial. Int..

[18] Fernstrom A., Hylander B., Rossner S. (1996). Taste acuity in patients with chronic renal failure. Clin. Nephrol..

[19] Van der Giet M., Tolle M., Pratico D., Lufft V., Schuchardt M., Horl M.P., Zidek W., Tietge U.J. (2010). Increased type IIA secretory phospholipase A(2) expression contributes to oxidative stress in end-stage renal disease. J. Mol. Med..

[20] Stegmayr B. (2014). Uremic toxins and lipases in haemodialysis: A process of repeated metabolic starvation. Toxins.

[21] Van Hall G. (2015). The Physiological Regulation of Skeletal Muscle Fatty Acid Supply and Oxidation During Moderate-Intensity Exercise. Sports Med..

[22] Florens N., Calzada C., Lyasko E., Juillard L., Soulage C.O. (2016). Modified Lipids and Lipoproteins in Chronic Kidney Disease: A New Class of Uremic Toxins. Toxins.

[23] Bagdade J.D. (1970). Uremic lipemia. An unrecognized abnormality in triglyceride production and removal. Arch. Intern. Med..

[24] Attman P.O., Alaupovic P., Tavella M., Knight-Gibson C. (1996). Abnormal lipid and apolipoprotein composition of major lipoprotein density classes in patients with chronic renal failure. Nephrol. Dial. Transplant..

[25] Bagdade J.D., Yee E., Wilson D.E., Shafrir (1978). Hyperlipidemia in renal failure: Studies of plasma lipoproteins, hepatic triglyceride production, and tissue lipoprotein lipase in a chronically uremic rat moedl. J. Lab. Clin. Med..

[26] Vaziri N.D. (2009). Causes of dysregulation of lipid metabolism in chronic renal failure. Semin. Dial..

[27] Vaziri N.D., Yuan J., Ni Z., Nicholas S.B., Norris K.C. (2012). Lipoprotein lipase deficiency in chronic kidney disease is accompanied by down-regulation of endothelial GPIHBP1 expression. Clin. Exp. Nephrol..

[28] Cheung A.K., Parker C.J., Ren K., Iverius P.H. (1996). Increased lipase inhibition in uremia: Identification of pre-beta-HDL as a major inhibitor in normal and uremic plasma. Kidney Int..

[29] Mahmood D., Makoveichuk E., Nilsson S., Olivecrona G., Stegmayr B. (2014). Response of angiopoietin-like proteins 3 and 4 to hemodialysis. Int. J. Artif. Organs.

[30] Zhang R. (2016). The ANGPTL3–4-8 model, a molecular mechanism for triglyceride trafficking. Open Biol..

[31] Ebert T., Kralisch S., Hoffmann A., Bachmann A., Lossner U., Kratzsch J., Bluher M., Stumvoll M., Tonjes A., Fasshauer M. (2014). Circulating angiopoietin-like protein 8 is independently associated with fasting plasma glucose and type 2 diabetes mellitus. J. Clin. Endocrinol. Metab..

[32] Baranowski T., Kralisch S., Bachmann A., Lossner U., Kratzsch J., Bluher M., Stumvoll M., Fasshauer M. (2011). Serum levels of the adipokine fasting-induced adipose factor/angiopoietin-like protein 4 depend on renal function. Horm. Metab. Res..

[33] Nasstrom B., Olivecrona G., Olivecrona T., Stegmayr B.G. (2003). Lipoprotein lipase during heparin infusion: Lower activity in hemodialysis patients. Scand. J. Clin. Lab. Investig..

[34] Nasstrom B., Olivecrona G., Olivecrona T., Stegmayr B.G. (2001). Lipoprotein lipase during continuous heparin infusion: Tissue stores become partially depleted. J. Lab. Clin. Med..

[35] Kessler M., Moureau F., Nguyen P. (2015). Anticoagulation in Chronic Hemodialysis: Progress Toward an Optimal Approach. Semin. Dial..

[36] Mahmood D., Grubbstrom M., Lundberg L.D., Olivecrona G., Olivecrona T., Stegmayr B.G. (2010). Lipoprotein lipase responds similarly to tinzaparin as to conventional heparin during hemodialysis. BMC Nephrol..

[37] Nasstrom B., Stegmayr B.G., Olivecrona G., Olivecrona T. (2003). Lower plasma levels of lipoprotein lipase after infusion of low molecular weight heparin than after administration of conventional heparin indicate more rapid catabolism of the enzyme. J. Lab. Clin. Med..

[38] Stegmayr B., Olivecrona T., Olivecrona G. (2009). Lipoprotein lipase disturbances induced by uremia and hemodialysis. Semin. Dial..

[39] Nasstrom B., Stegmayr B., Gupta J., Olivecrona G., Olivecrona T. (2005). A single bolus of a low molecular weight heparin to patients on haemodialysis depletes lipoprotein lipase stores and retards triglyceride clearing. Nephrol. Dial. Transplant..

[40] Stegmayr B.G., Jonsson P., Mahmood D. (2013). A significant proportion of patients treated with citrate containing dialysate need additional anticoagulation. Int. J. Artif. Organs.

[41] Kozik-Jaromin J., Nier V., Heemann U., Kreymann B., Bohler J. (2009). Citrate pharmacokinetics and calcium levels during high-flux dialysis with regional citrate anticoagulation. Nephrol. Dial. Transplant..

[42] Brandl M., Strobl K., Hartmann J., Kellner K., Posnicek T., Falkenhagen D. (2012). A target-orientated algorithm for regional citrate-calcium anticoagulation in extracorporeal therapies. Blood Purif..

[43] Chazot C., Shahmir E., Matias B., Laidlaw S., Kopple J.D. (1997). Dialytic nutrition: Provision of amino acids in dialysate during hemodialysis. Kidney Int..

[44] Ikizler T.A., Flakoll P.J., Parker R.A., Hakim R.M. (1994). Amino acid and albumin losses during hemodialysis. Kidney Int..

[45] Liu Y., Xiao X., Qin D.P., Tan R.S., Zhong X.S., Zhou D.Y., Liu Y., Xiong X., Zheng Y.Y. (2016). Comparison of Intradialytic Parenteral Nutrition with Glucose or Amino Acid Mixtures in Maintenance Hemodialysis Patients. Nutrients.

[46] Blumenkrantz M.J., Gahl G.M., Kopple J.D., Kamdar A.V., Jones M.R., Kessel M., Coburn J.W. (1981). Protein losses during peritoneal dialysis. Kidney Int..

[47] Rigalleau V., Gin H. (2005). Carbohydrate metabolism in uraemia. Curr. Opin. Clin. Nutr. Metab. Care.

[48] Czabak-Garbacz R., Schneditz D., Zierler E., Eichmann E., Harter G., Hafner-Giessauf H., Obermayer-Pietsch B. (2011). Blunted insulinemia using high dialysate glucose concentration during hemodialysis. ASAIO J..

[49] Raimann J.G., Kruse A., Thijssen S., Kuntsevich V., Dabel P., Bachar M., Diaz-Buxo J.A., Levin N.W., Kotanko P. (2012). Metabolic effects of dialyzate glucose in chronic hemodialysis: Results from a prospective, randomized crossover trial. Nephrol. Dial. Transplant..

[50] Yamagishi S., Matsui T. (2016). Pathologic role of dietary advanced glycation end products in cardiometabolic disorders, and therapeutic intervention. Nutrition.

[51] Wathen R.L., Keshaviah P., Hommeyer P., Cadwell K., Comty C.M. (1978). The metabolic effects of hemodialysis with and without glucose in the dialysate. Am. J. Clin. Nutr..

[52] Limkunakul C., Sundell M.B., Pouliot B., Graves A.J., Shintani A., Ikizler T.A. (2014). Glycemic load is associated with oxidative stress among prevalent maintenance hemodialysis patients. Nephrol. Dial. Transplant..

[53] Arsov S., Graaff R., van Oeveren W., Stegmayr B., Sikole A., Rakhorst G., Smit A.J. (2014). Advanced glycation end-products and skin autofluorescence in end-stage renal disease: A review. Clin. Chem. Lab. Med..

[54] Meerwaldt R., Hartog J.W., Graaff R., Huisman R.J., Links T.P., den Hollander N.C., Thorpe S.R., Baynes J.W., Navis G., Gans R.O. (2005). Skin autofluorescence, a measure of cumulative metabolic stress and advanced glycation end products, predicts mortality in hemodialysis patients. J. Am. Soc. Nephrol..

[55] Graaff R., Arsov S., Ramsauer B., Koetsier M., Sundvall N., Engels G.E., Sikole A., Lundberg L., Rakhorst G., Stegmayr B. (2014). Skin and plasma autofluorescence during hemodialysis: A pilot study. Artif. Organs.

[56] Ramsauer B., Engels G., Arsov S., Hadimeri H., Sikole A., Graaff R., Stegmayr B. (2015). Comparing changes in plasma and skin autofluorescence in low-flux versus high-flux hemodialysis. Int. J. Artif. Organs.

[57] Ramsauer B., Engels G.E., Graaff R., Sikole A., Arsov S., Stegmayr B. (2017). Skin- and Plasmaautofluorescence in hemodialysis with glucose-free or glucose-containing dialysate. BMC Nephrol..

[58] Abe M., Kalantar-Zadeh K. (2015). Haemodialysis-induced hypoglycaemia and glycaemic disarrays. Nat. Rev. Nephrol..

[59] Jiang J., Chen P., Chen J., Yu X., Xie D., Mei C., Xiong F., Shi W., Zhou W., Liu X. (2012). Accumulation of tissue advanced glycation end products correlated with glucose exposure dose and associated with cardiovascular morbidity in patients on peritoneal dialysis. Atherosclerosis.

[60] Makulska I., Szczepanska M., Drozdz D., Polak-Jonkisz D., Zwolinska D. (2013). Skin autofluorescence as a marker of cardiovascular risk in children with chronic kidney disease. Pediatr. Nephrol..

[61] Siriopol D., Hogas S., Veisa G., Mititiuc I., Volovat C., Apetrii M., Onofriescu M., Busila I., Oleniuc M., Covic A. (2015). Tissue advanced glycation end products (AGEs), measured by skin autofluorescence, predict mortality in peritoneal dialysis. Int. Urol. Nephrol..

[62] Macsai E., Benke A., Kiss I. (2015). Skin Autofluorescence and Mortality in Patients on Peritoneal Dialysis. Medicine.

[63] Macsai E., Benke A., Cseh A., Vasarhelyi B. (2012). Factors influencing skin autofluorescence of patients with peritoneal dialysis. Acta Physiol. Hung..

[64] Crowley L.E., Johnson C.P., McIntyre N., Fluck R.J., McIntyre C.W., Taal M.W., Leung J.C. (2013). Tissue advanced glycation end product deposition after kidney transplantation. Nephron Clin. Pract..

[65] Cano N.J., Fouque D., Roth H., Aparicio M., Azar R., Canaud B., Chauveau P., Combe C., Laville M., Leverve X.M. (2007). Intradialytic parenteral nutrition does not improve survival in malnourished hemodialysis patients: A 2-year multicenter, prospective, randomized study. J. Am. Soc. Nephrol..

[66] Sundell M.B., Cavanaugh K.L., Wu P., Shintani A., Hakim R.M., Ikizler T.A. (2009). Oral protein supplementation alone improves anabolism in a dose-dependent manner in chronic hemodialysis patients. J. Ren. Nutr..

[67] Gutierrez A., Bergstrom J., Alvestrand A. (1994). Hemodialysis-associated protein catabolism with and without glucose in the dialysis fluid. Kidney Int..

[68] Hynote E.D., McCamish M.A., Depner T.A., Davis P.A. (1995). Amino acid losses during hemodialysis: Effects of high-solute flux and parenteral nutrition in acute renal failure. J. Parenter. Enteral Nutr..

[69] Gutierrez A. (1996). Protein catabolism in maintenance haemodialysis: The influence of the dialysis membrane. Nephrol. Dial. Transplant..

[70] Gil H.W., Yang J.O., Lee E.Y., Lee E.M., Choi J.S., Hong S.Y. (2007). The effect of dialysis membrane flux on amino acid loss in hemodialysis patients. J. Korean Med. Sci..

[71] Umber A., Wolley M.J., Golper T.A., Shaver M.J., Marshall M.R. (2014). Amino acid losses during sustained low efficiency dialysis in critically ill patients with acute kidney injury. Clin. Nephrol..

[72] Mineshima M. (2015). The past, present and future of the dialyzer. Contrib. Nephrol..

[73] Sterky E., Stegmayr B.G. (2005). Elderly patients on haemodialysis have 50% less functional capacity than gender- and age-matched healthy subjects. Scand. J. Urol. Nephrol..

[74] Jones M., Hagen T., Boyle C.A., Vonesh E., Hamburger R., Charytan C., Sandroni S., Bernard D., Piraino B., Schreiber M. (1998). Treatment of malnutrition with 1.1% amino acid peritoneal dialysis solution: results of a multicenter outpatient study. Am. J. Kidney Dis..

[75] Jones M.R., Gehr T.W., Burkart J.M., Hamburger R.J., Kraus A.P., Piraino B.M., Hagen T., Ogrinc F.G., Wolfson M. (1998). Replacement of amino acid and protein losses with 1.1% amino acid peritoneal dialysis solution. Perit. Dial. Int..

[76] Burmeister J.E., Miltersteiner Dda R., Burmeister B.O., Campos J.F. (2015). Risk of hypoglycemia during hemodialysis in diabetic patients is related to lower pre-dialysis glycemia. Arch. Endocrinol. Metab..

